# Repertory of the Back

**Published:** 1898-01

**Authors:** E. H. Wilsey

**Affiliations:** Parkersburg, W. Va.


### REPERTORY OF THE BACK.

E. H. Wilsey, M. D., Parkersburg, W. Va.

(Continued from page 493.)

**Eruption** on back, aching like a sore, Bry.
— on back like zoster, Cist-c.
— of pimples on back, Dig.
— of backache, with suppressed eruption, Psor.
— pustular on back, Aurm-nat.
— much itching, with eruption on back, Bar-c.
— itching on upper part of back, Lyc.

**Erysipelatous.** Broad belt of erysipelatous inflammation extending across from point of right shoulder to upper part of left arm, Apis.
— inflammation extending from back like a girdle around abdomen, Merc.

**Evening.** Drawing in back while sitting down in evening, Tereb.
— chilliness over back nape and occiput toward evening for ten days, with a sensation like horripilation, Dulc.
— drawing pain in back in evening, Nit-ac.
— pain in back in evening, extending down thighs and legs, Still.
— pains in back, worse evenings, Naja.
— bruised pain in small of back from morning till evening, better by going to bed, Nat-s.
— insupportable backache toward evening and on arising from a seat, Ars.

**Evacuation.** Stitch in small of back, better by an evacuation, Indigo.

**Excoriated.** The skin on left side of back pains as if excoriated. Petrol.

**Exertion.** Pain, as after excessive exertion in back, Chel.

**Exhaustion.** As after hard work, especially in back, Apis.

**Extending.** From other parts to back, pains, Sep.

**Faint** feeling while standing in back, Sep.

**Fall.** She feels as if the muscles of back were not strong enough to support the body which always tends to fall forward, Am-c.

**Fall.** Pain as from a, on dorsal vertebræ, Ruta.

— pain in back, as after a fall, KALI-C.

**Falling.** Unable to hold urine after being struck on back by falling from a wagon, HYPER.

**Fatigue.** Tensive pressure, like severe fatigue, from right scapula, extending into side of back, even to sacrum, also early in bed, but especially while driving, Kali-c.

— pain in small of back, as from fatigue, Aur-m.

— pain in back, as from fatigue, especially after eating, and while sitting, Ant-t.

**Feet.** Backache extending into feet, Borax.

— lies in bed on back with feet drawn up, PULS.

— lies on back with feet crossed, Rhod.

— violent cramp in back and in feet, Iod.

— shuddering over back extending into feet and arms, Calc-ars.

— chill in back at 3 P. M.; still worse in evening after lying down for a quarter of an hour, with cold feet, without any heat or sweat following, Lyco.

— pain in small of back while lying on back, with great weariness going down to feet, LYCO.

**Fever.** Intermittent, with pain in small of back, LACH.

**Finger.** Painful stiffness of small of back and of finger-joints, which she could hardly bend, MANG.

**Flashes** up back, with stool, Podo.

**Flatus.** Drawing in lower part of back, as from incarcerated flatus, Nat-c.

— pain in small of back, with incarcerated flatus, Amm-m.

— pain like a drawn and beaten sensation in back then extending into small of back, thence to abdomen, where there is flatulence with pain, then discharge of flatus and leucorrhœa, Caustic.

— pain in back, as from incarcerated flatus, Amor-s., Calad., CALC-C.

— severe pain in small of back from within outward, as if bruised; pain appears at 5 A. M., and is better by moving about, and by passing flatus, Ruta.

**Fleas**. Crawling in back, as from fleas, Pallad.

**Flesh**. Sensation as if flesh was torn loose on lower part of back, Lyc.

**Flushes** of heat or currents of cold air down back, Cof-crud.

**Formication.** In back, Phos., Nat-c., Rhod.

— up and down left half of back, Euony.

— in back and small of back, Phos-ac.

**Fracture**. Tearing and pain, as from a fracture in scapula, with stiffness of back and nape, Nat-m.

**Fright**. An undulating shock in back above the left ileum, causing him to start with fright, Stann.

**Furuncle** on back, Caustic, Zinc., Graph. (See boils.)

**Fullness** in back after eating, Cham.

**Gaping.** Drawing in back with gaping, stretching and bending backward; worse evening and morning on getting awake and moving about, Cal-ph.

**Genitals**. Spasmodic drawing from behind forward into genitals or down into thighs, Kreos.

— great pain in back, sometimes extending through abdomen to front and into genitals, Cham.

**Girdle**. Sensation in small of back and about hips while stooping, as from a girdle, Gentia.

— erysipelatous inflammation extending from back like a girdle around abdomen, Merc.

**Gliding**. Sensation as if the vertebræ were gliding over one another when turning in bed, Sul.

**Glow** on back after chilliness, Ars-hyd.

**Gnawing** pain in small of back, Sul., Nicol.

— pain in back, Alumina.

— pain in small of back and hips, going thence to abdomen and back again, both in rest and in motion, Amm-c.

— in A. M. and night, all her limbs ache with a gnawing pain in small of back, more while at rest than when in motion, Amm-c.

— about an hour after eating dinner drawing pain in stomach and a gnawing extending to back, where it is most acute, then great exhaustion and lassitude, Sep.

**Gnawing,** violent pain in small of back like a gnawing, drawing up between the shoulders, where it beeomes so violent she feels like weeping, Alum.

— in back, Agari., Alum., Amm-c.

— in lower part of back, worse after going to bed, Naja.

— tearing down back, with feeling in bones as if dogs were gnawing them, Nat-s.

— pressing and tearing in back while walking after rising from a seat, Canth.

— severely itching rash on the nape, the back and thighs, always worse and more gnawing after scratching, with pricking as from needles, Mez.

**Grasping,** pinching in stomach, which contracts the chest, with grasping together of back, awakening her in morning from sleep, Con.

— attacks twice daily, at first a drawing in back like a grasping in dorsal, sides below ribs extending around into stomach, where it turns about and disappears as with an eructation, Nit-a.

**Griping,** now in dorsal and now in abdominal muscles, Peonia.

— in right side of back, Sil.

— in back during menses, CALC-C.

— contraction in back with sensation of coldness, griping, and sore feeling, Con.

— pressing in back opposite bowels, then oppressive stitches on least motion or breathing, so that he had to walk bent, when standing still a griping as from a malignant ulcer and with oppression of chest, Arg-m.

— and twisting in small of back, as from tongs, then pain in arms and feet, as if they would turn outward, Graph.

**Groins,** pains in both groins and back, Lyss.

— pain in small of back, going to groins and down limbs, Plat.

**Grumbling** and throbbing sensation in lower part of small of back, Bar-c.

**Hammer,** when stooping a sudden pain in back as if struck by a hammer, better by pressing back against something hard, Sep.

**Hard** pain in back, is better by lying on something hard, Nat-m.
**Head,** pain in back of head and limbs, Merc-c.
**Headache,** pain in small of back at 6 p. m., with headache, Cobalt.
— and backache, Lyss., Cina., Phyto., Kali-c.
— as if cold water was poured down back, with headache, Alum.
— and weak back in the morning, Pallad.
**Heart,** lying on back relieves pain in heart, Tell.
— beats quickly and strongly when lying on back, Ars.
— a sinking sensation going from heart to back, Lyss.
— sticking in back opposite heart, Spig.
**Heart–burn,** burning in back near last ribs, right side, like heart-burn, Lyss.
**Heat** in general in back, Glon., Dulc., Phos., Aco., Podo., Alum., Berb., Camph., Calc., Nitr-sp-d., Ver-v., Agari., Cocoa, Merc-per., Coff., Ars-h., Apis, Hyos., Med., Sarrac.
— and burning like burning heat on back, Apis.
— frequent rising of heat from the back into the head, with redness of face in p. m. while sitting, Phos.
— feeling of intermittent heat rising up back, Phos.
— much heat in lower part of back in renal region, Phos.
— cannot tolerate any heat near back, Phos.
— sensation of heat all over body especially on thighs and on back, Stann.
— extending from neck down back, Paris-q.
— back is cold while there is dry heat in sacrum and thighs, Sul.
— flushes of heat or currents of cold air down back, Cof-cr.
— burning in left chest through to back with cough, Oleum-jec.
— sudden on whole back while sitting down and soon after followed by perspiration, with very much contracted pupils, Mang.
— sensation of heat down the back in the morning, Con.
— after sensation of heat tearing pains across small of back, with fullness of joints of legs, extending to all parts of body, Ham.

**Heat,** a thrill of heat with drawing pain from nape of neck over back, Nat-c.

— pain and beating in small of back, Lac-can.

— sensation of heat in the whole body especially in the back, where she imagined she perspired, Zinc.

— at night, first sweat on back, causing him to wake up at 4 o'clock then dry, internal heat, with uncomfortableness so that he cannot go to sleep again, Petrol.

— on cheeks and flushes of heat on back in the evening while walking in open air, Phos-ac.

— in face and perspiration on forehead, with heat on chest and back, combined with needle pricks from within outward, most frequent and severe in the neck, Sarsa.

— aching in middle of back with flashes of heat, Lappa.

— flashes of heat in back, Merc-peren.

— violent fever with chill in back; he cannot get warm, and yet has internal heat, Nit-ac.

**Heaviness** in back; worse by sitting; better by motion and pressure, ALOE.

— in morning almost as if asleep, SEP.

— in back in morning as if he had lain in wrong position, and weariness if he had not slept enough, Sul.

— in small of back, Coloc., Pic-ac.

— and pressure in small of back, as if one had received a blow while sitting, RHUS-T.

— in back with tightness of chest, Carbo-v.

— drawing in back with heaviness of arms, Carbo-v., Ant-t.

— in back, Petrol.

— and weariness in back while lying down, Phos.

— in back with dyspnœa, NAT-M.

— in back in morning on waking, as if she could not turn nor raise herself, or as if she had lain in wrong position, almost as if parts had gone to sleep, Sep.

— and weight in back from womb and ovaries, CON.

— of head, with weakness of cervical muscles, COCCUL.

— pressure in small of back, with leaden-like heaviness of lower limbs, CAMPH.

**Heaviness** in back in morning on rising and in lower limbs, Sul., Sep.

— feeling and stiffness in lower part of back, Zing.

— in back; better by expulsion of gas, Berb.

**Hemorrhage.** Pain in back, with uterine hemorrhage, Trill.

**Hemorrhoidal.** Lame back, with very painful, dark-purple, hemorrhoidal tumors, ÆSC-GLAB.

**Herpetic** spots on back and chest, itching in evening, Sul.

— small herpetic itching spots on both sides of the neck and on small of back, Lyco.

**Hips.** Pain in small of back and stitches in both hips and in left chest at night, LYC.

— pain in small of back extending to right hip, Pallad.

— pain in back just above hips, NUX-M.

— pain in back and hips and dreads insanity, Lil-tig.

**Horripilation.** Chilliness over back, nape, and occiput, toward evening for ten days (with a sensation like horripilation), Dulc.

**Hot** sensation down back, GLON, Sul.

— sponge near back causes him to wince, PHOS.

— water, internal chills for several P. M.'S, for half an hour or so, and at times sensation as if hot water was in scrobiculus cordis and in the back, Phos.

**Humped.** Sensation as if she would be hump-backed if she did not wear stays to support weak feeling in middle of back, Raph.

**Ice.** Drawing in limbs and back with great coldness even in back as if she lay on ice for two hours, Lyc.

**Icy** cold hands and coldness in back, CACT.

— chill from 6 to 7 P. M. with icy cold feeling on back, she found it hard to get warm, Mur-ac.

— first some chilliness down back, with icy cold hands, then intense heat, with distension of abdomen, Sil.

— in back between shoulders in a spot where former pain had been only internally and not to be warmed either by feathers or wool; after half a day the cold turns into itching, Amm-m.

**Incarcerated.** Pain in small of back, could hardly rise from seat, with watery, bloody discharge from vagina; from incarcerated flatus, CALC-C.

**Incurvation** and swelling of vertebræ of neck and back, CALC-ARS.

**Inflamed.** The left side of the back is painful as from pressure on inflamed spot, Nat-m.

**Inflammatory** pains intense all through back, OX-ac.

**Inflated.** Pressure at night in the internally inflated abdomen, worse by other than position on back, with compressed breath and quick pulse, Mez.

**Injury,** stiffness on back as from an injury, PRUNUS-SP.

**Inspiration,** pain in eighth dorsal vertebræ and in back under short ribs; worse by motion and deep inspiration, LOB-C.

— rheumatic tension in back and in right side of chest; stronger on inspiration, Lyc.

**Internal** chill for several P. M.'s for half an hour or so and at times sensation as if hot water was in the scrobiculus cordis and back, Phos.

**Intolerable** sharp pains rendering motion when not in an erect position intolerable, Tereb.

— pain commencing early in morning before arising, in lower part of back, right side, extending to gluteal region and lasting all day, Cactus.

**Inward,** pain in back which makes him bend inward, with heat in evening disappearing in morning, Jambos.

**Itching,** burning and sticking pain in small spot of back; worse after scratching, Jambos.

— and burning pain in back, Mag-m.

— shooting pain in middle of back, ceasing when rubbed, Mang.

— severe itching pain in small of back; worse from motion, COLOC.

— intense in back, and pain after scratching, NIT-AC.

— acne on back, Carbo-an.

— all over body, face, hands, hips, abdomen, back, and feet, SEP.

— all over back, Nit-ac.

— pimples on small of back, Niccol.

— in middle of back; better by rubbing, Mang.

**Itching,** small dry red pimples on back, breast and arms, at first itching, Iod.

— quickly arising here and there on the body, back, arms and pubic region, and even on the scalp, Phos-ac.

— stitches on back, Sul.

— severe on back day and night, Bar-c.

— on small of back and between nates; must scratch it raw, BAR-C.

— and tingling formication in whole back, Nat-c.

— tickling on whole back, Agari.

— violently on back, Lyco., Nit-ac. (for fourteen days ANT-CR.)

— on walking sticking at every breath, Sul.

— on back when undressing, NAT-S.

— violent on back, lower limbs and nates in evening in bed, with wheals after scratching, which soon pass off again, Lyco.

— on back and thighs she must scratch; Nat-m.

— and red blotches on back, Asclep-tub.

— and burning in back, Agari.

— and burning on face, on the back, and on the head, Kali-c.

— burning down back, Daphne.

— much on back and on calves, CAUST.

— on back and shoulders as from crawling insects evenings on going to bed, Osmium.

— by day on back and arms, Graph.

— in right dorsal muscle, Spig.

— corroding in back by day, Guaj.

— in back, Sil., Sul.

— herpatic spots on back and chest in evening, Sul.

— over right hip in back, Iod.

— on back, Spong., Cist-can.

— on back edge of shoulder and nape of neck, Therid.

— and perspiration on back, Phos-c.

— of the back and sometimes perspiration, Caust.

— eruption of pimples on back with itching in evening in bed, Nat-m.

— and itching pimples on back, Calc-c.

-—violent on back toward neck, Lyco.

**Jerks**. Spasmodic, painful jerks in left side of back, Agari.

**Jerking**. Violent pain in small of back in raising the thigh when sitting, Agari.

— painful in small of back on various motions, Petrol.

— in muscles of left side of back, Carbo-v.

— head backward while lying on back, HYPERI.

— drawing in cervical muscles near nape of neck, extending downward in evening while lying on opposite side, Viola-odo.

— sensation in back and nape extending toward head, Nat-m.

— tearing in small of back, especially on moving, Alum.

— in back and left arm with vertigo, blackness before eyes and falling down, Diadema.

— tense feeling in small of back, worse going up stairs, with occasional jerking toward hip-joint, Carboneum-s.

— like electric shocks in back, Ang.

— in muscles between shoulders; better moving shoulders up and down, Ran-b.

— stitch-like in small of back when walking, extending toward hip rather than upper part; gets worse by sitting or standing, FER.

**Knee**. Weakness of knees and legs, with pain in small of back, NUX-M.

— lies on back with knees drawn up, MERC-C.

— deep shooting in cardiac region, extending to axilla and back, with shooting in thigh, extending to the knee when sitting, ceasing on rising up in evening, Mur-ac.

**Kink**. Sudden catch or kink in back, SEC.

**Knife**. Lancinating stitches, as from a sharp knife in back through loins, extending to legs, IGN.

— in middle, between right loin and spine; intermittent and deep, sharp knife-like stitches, quite internal to intestines, Verbas.

**Knives**. Violent stabbing, as with knives, between shoulders; it awakes her out of sleep, shortening her breath; it appears while she is lying on her back, and is better by lying on right side, Nitrum.

**Knocked**. Feels as if small of back had been knocked away, Arg-m.

**Labor.** Intense pain in back while sitting and worse when lying, and not while walking or performing manual labor, Nat-m.
— like pains in small of back and sacral region, urging to urinate and ineffectual desire to stool, KREOS.
— pain in small of back like labor pains, Eup-per., PULS.
— complains of sore pain in back during labor; cries, "Hold my back! Oh! hold my back!" CAUSTIC.
— sharp pain in small of back, with very acute labor-like pains running through to front at intervals of a few minutes, occasional shooting down gluteal region, KALI-C.
— backache after labor, HYPERI.
— backache during labor, Chlorof.
**Lacerated.** Burning, tearing, drawing, or bruised pain in back, NUX-V.
— drawing or boring pain in back, Coccul.
**Lacerating** and sharp stitches in small of back, Dig.
— and drawing in back, Opium.
**Lame.** Sensation as if lame in small of back, NUX-V.
— and stiff; weakness in small of back, Diosc.
— and sore feeling in small of back, LEPT.
**Lameness.** In back, Conval.
— in back; cannot sit up without support, Diosc.
— in small of back, as from a cold, DULC.
— in small of back, with leucorrhœa, CAUSTIC.
— paralytic or rheumatic lameness and stiffness of back after rest; better by gentle motion, KALI-PHOS.
— a sensation of lameness in back, evenings in bed, KALMIA.
— and weakness in small of back and subsequent lassitude, Con.
— in back, hips and legs, Fluor-ac.
— of back from walking or standing in lower part, Zing.
— tired lameness of legs, with backache, KALI-BROM.
— and stiffness of back, worse from motion, better during rest, returns after sitting awhile, CUP-ARS.
— and painful feeling in back, HYPER.
— all limbs and back pain, with lameness, Meph.
**Laming.** Tearing, drawing pains in back and thighs, CHINA.
— pain in small of back while sitting or standing, COF-CR.

**Lancinating.** Sharp or cutting pain, running up back, with constriction of arms, COLOC.

— a severe, pressive pain in back, under right scapula, which at inspiration changes into a lancinating pain, Cup.

— pains and weight in back and over part of abdomen, extending from womb and ovaries, Con.

— pain in back, extending to right scapula, Lyc.

— pain in back while walking, Sul.

— in small of back in a transverse direction, Cup.

**Lassitude** and pain in back, as if bruised, in region of short ribs, with ill humor, RAN-B.

— painful along back, with bruised feeling down to sacrum, Ginseng.

— weak, with pain in back, and pressing headache mornings; painful lassitude in small of back and lower limbs, Coloc.

— weakness and lameness in small of back, Con.

— dragging in back and lumbar muscles, with lassitude on exercising in open air, Tereb.

**Lascivious.** When lying on his back he has lascivious dreams and seminal emissions without erections, Colocy.

**Laugh.** Cannot laugh on account of pain in small of back, Phos.

**Laughing.** Aching in small of back, worse either by coughing or laughing. Tell.

— after falling from a height upon back, acute pain in lower part of back or right side when laughing, sneezing or a quick breath, CON.

**Leaning,** throbbing in small of back; worse by sitting upright, better leaning back, Sepia.

— difficult to sit straight without leaning against something, as back is very weak, Agari.

**Left,** stitches in left side of back, Hepar.

**Legs**, drawing intolerable in small of back and down into legs, especially noticed in the ischia, frequently in evening, LACH.

**Leucorrhœa** with backache, PULS., MUR-AC.

— with lameness in small of back, CAUSTIC.

— with backache alternating with catarrh, KALI-B.

— pain in small of back accompanying leucorrhœa, KALI-FER.

**Leucorrhœa,** drawing in back and as if bruised, thence the pain extended to the sacrum and abdomen, where much flatus accumulated with bellyache, and as the flatus was discharged leucorrhœa appeared, Caustic.

**Lie,** pains as if crushed in back, so she could not lie on it at night, Amm-m.

— painful on left side of back so he could not lie on it for three nights, Carbo-a.

— cannot lie for any length of time on back, Hyper.

— could not lie upon back or either side, GLON.

— on back in typhoid, ZINC.

— on back with mouth open, LACH.

— cannot lie on back in cardiac rheumatism, Phos.

— if he lies on his back it aggravates cough, Eup-per.

— inclination to lie on his back in consumption, FER-IOD.

— on his back with open staring eyes, STRAM.

— on back with head low, prefers to, DIG.

— impossible for him to stoop or lie upon his back, Tereb.

— on back with knees drawn up, MERC-C., HELL., Tarant.

— violent suppurative pain in small of back at night so she can hardly lie on her right side, better mornings after getting up, Nat-s.

**Lies** on back with one leg stretched and other drawn up (in pregnancy), Stann.

— on back with legs turned on stomach, Puls.

— on back in pneumonia, Puls.

— on back and all symptoms are better, Uva-ursi.

— on his back always when he dreams at night, Mang.

— on back with feet crossed, Rhod.

—on his back when sleeping with one hand under his occiput, the other arm above his head, Coloc.

**Lifting.** Pain in small of back after heavy lifting and taking cold at same time, SUL.

— sudden acute pain in small of back while lifting a light board, so that he could raise himself only with difficulty, RHUS.

— sudden stitch in back while lifting, cannot move without great pain, SEPIA.

**Lifting.** Pain in small of back as after over-lifting, worse at rest at night and in morning; also, when rising from a seat, STAPH.
— pains in back after lifting, begins in lower limbs and vertebræ and extends to hypochondrium, NUX-V.
**Lightning**-like pains about small of back so that he cannot move in evening, sticking when sitting, Nat-c.
— like pains of a sticking character in back, NIT-AC.
**Limbs** pain and backache; worse in warm room toward evening, better in cold open air, KALI-S.
— much pain in back and limbs at same time, Ip.
— pain runs through back into limbs repeatedly, Camph.
— tearing in limbs with backache, RHUS.
**Liver.** Pain in liver through to back, KALI-C.
— pain in back and right side from congestion of liver, LYC.
— pain from region of liver to back and shoulders, Ars-m.
**Load.** Tensive pain in small of back as from a heavy load or after long stooping, CHINA.
**Loins.** Coldness in small of back and loins, CAMPH.
**Looking.** Cracking sound from first cervical vertebræ on looking up in evening, Zing.
**Low.** Pain in back, sides and low down, PULS.
**Lower.** Considerable pain in lower part of back with a soreness felt through to pubic region; pressing finger on neck increases pain, LYSS.
— pain in lower part of back and spine between hips, Calab.
— pain in small of back and lower limbs, Bad.
— pain in lower part of back, Carbo-a.
**Lungs.** Pain through from back to front of both lungs and *vice versa*, Diosc.
— heavy pain in left lung, extending through to back and becoming stabbing, Papaya-vul.
**Lying.** Bruised pain in back from 4.30 P. M. till lying down at 9 P. M., Mag-c.
— pain in small of back while lying on back with great weariness, going down to feet, LYC.
— great pain in left side of back on lying down, Bar-c.

**Lying.** Bruised pain while lying on back, Hyosc.
— respiration difficult, especially while on back, in goitre, IOD.
— at night, on waking he always finds himself lying on his back, SUL. Mur-ac.
— when lying in bed on his back, music in his ears like the far-off humming of a bag-pipe, ceases on rising, but soon recurs on lying down, Nat-c.
— if lying on side straightens out on back quick as lightning, Cic.
— pain in small of back at night when lying on it, CHINA.
— on his back with left arm over his head a half conscious sleep, Dig.
— on back worse, SPIG.
— on his back heart beats strongly, Ars.
**Meningitis.** Darts in small of back, abuse of mercury, Kali-iod.
**Menses.** Bearing down in small of back as if menses would come on, APIS.
— great pressure from within out during menses, NUX-M.
— burning in small of back, especially during a delay of menses, Phos.
— aching at 10 A. M. during menses, then weakness of thighs, Am-m.
— in evening coolness in back, awaking after midnight with cramp and coolness of stomach, which continues till about noon, Kali-c.
— pain in small of back instead of menses, SPONG.
— pain in back as before menses, Bar-c., Berb., HYDRAS., LYC., PULS., SPONG.
— pain in back labor-like before menses, DIG.
— pains in back before menses, commence in and go around, ending in cramps in uterus, VIB-O.
— labor-like pains in back during menses, Agar., CALC., NIT-A., SUL.
— dreadful pain in back during menses, SARSA.
— during menses prolapsus uteri, AMM-M.
— pain in back with scanty menses, LAC-DEF.
— pain in back from suppressed menses, PULS.
— pain in back every few minutes with menses, USTIL.

**Menses**. Pain in back worse during menses when walking, MAG-M.
— tearing pain in back during menses, CAUST.
— pain in small of back during menses, Iod., Ratan.
— a great deal of pain in back as if menses would come on, Coccul.
— intermittent pain in back during menses, Mag-c.
— drawing pain in back during menses; better stooping, worse stretching, Mag-c.
— pain as if back would break during menses, VIB-O.
— pain in back and loins and hypogastrium as if menses were coming on; worse early part of evening in close room, better in open air and moving about, VIB-OP.

